# Book Notices

**Published:** 1885-05

**Authors:** 


### BOOK NOTICES.

CHOLERA EPIDEMICA; HOMŒOPATHIC TREATMENT. By Benjamin Ehrman, M. D. Cincinnati.

In a little pamphlet, Dr. Ehrman gives his experience in treating this disease and the indications for the remedies he used. For the stage of *collapse,* Dr. Ehrman recommends:

CARBO VEG., for anxiety of mind, cold breath and tongue, choleric face, cold, clammy sweat, hoarseness, suppressed pulse and urine, desire for cold air, aversion to warm coverings, and general prostration. Give 30th potency.

SECALE COR., for the following condition: the patient lies quietly in a semi-comatose or semi-paralyzed state, complains in a husky whisper of dimness of vision, dullness of hearing, tingling in the ears; also for internal heat, or burning with external coldness, numbness, and formication in limbs, with cold, clammy sweat, suppressed pulse and urine, and *aversion* to being *covered.* A great many cases presenting the above symptoms, given up by other physicians, were cured with the 30th potency in a short time.

HYDROCYANIC ACID. This remedy was only once employed by me in a desperate case, but with success. A lady, aged forty-four years, was attacked with cholera, after having had a miscarriage and flooding for a week, and was attended by the founder of the Physio-Medical school until collapse set in, when he gave her up, and I was called. The principal feature of the case was, in addition to the general prostration and extreme emaciation, THREATENING PARALYSIS OF THE HEART. In consideration of the teas and herbs just used, ten drops of the 1st dilution was dissolved in a glass half full of water, of which one teaspoonful was given every fifteen minutes for the first hour, and afterward every thirty minutes until better. Contrary to mine and to the friend's expectations, the patient was relieved in four or five hours, and finally restored by China and Phos. acid.

ACONITE. In order to have a clear perception when and how to use this remedy in collapse, and also to appreciate the importance of making at once a right selection of a remedy, the history of a clinical case will illustrate better than the bare symptoms merely could do. I was called late in the evening to a young lady, who was treated all day by three (allopathic and eclectic) physicians successively, but not successfully, and eventually given up by them all. When I arrived there I met one of them at the bedside of the patient. I said to him that I did not wish to interfere, if he was still attending, but he answered that he had done all he could, and that if I could restore the pulse again to this lady, he would himself beg me to do so.

The prominent symptoms were, besides general prostration, COMPLETE SUPPRESSION OF PULSE, VOICE, AND URINE, ICY COLDNESS OF THE LIMBS, restlessness, with *frightened* and *anxious* looks, etc. For this condition the remedy was selected; but in consideration of the heroic drugging, I decided to give eight or ten drops of the 1st dilution in a glass of water, one teaspoonful of which was to be given every fifteen minutes for the first hour, and afterward every half hour until better. Next morning I found the patient much better, pulse and warmth restored, and in a few days she was entirely well.

NICOTIN. PERFECT COLLAPSE, where diarrhœa, vomiting, and thirst had ceased entirely, ICY COLD PERSPIRATION on the *forehead*, and impending paralysis of the heart.

The 30th potency should be used. Direction: Give one dose every fifteen or thirty minutes for a few hours; afterward every one, two, or three hours, according to circumstances, until better.

### REPERTORY TO ECZEMA. By C. F. Millspaugh, M. D., Binghamton, N. Y. Pp. 43; price, 25 cents. New York: A. L. Chatterton Publishing Company, 1885.

Dr. Millspaugh is well known to the profession through the beautiful plates he is giving us of "American Medicinal Plants;" in this little repertory, the Doctor shows that he knows the therapeutic *uses* of our medicines as thoroughly as he does their botanical appearances. The repertory is printed only on one side of the page, leaving ample room for additions.

